# Sarcoid- like Phenomenon - ustekinumab induced granulomatous reaction mimicking diffuse metastatic disease: a case report and review of the literature

**DOI:** 10.1186/s13256-019-2137-1

**Published:** 2019-07-30

**Authors:** Mohamed M. Gad, Najdat Bazarbashi, Manpreet Kaur, Amit Gupta

**Affiliations:** 1 Cleveland Clinic, Cleveland, OH USA; 2 University Hospital Cleveland Medical Center, Case Western Reserve University, 11100 Euclid Ave, Cleveland, OH 44106 USA

**Keywords:** Sarcoid-like reaction, Sarcoidosis, Ustekinumab, Refractory psoriasis

## Abstract

**Background:**

The utilization of monoclonal antibodies has become more widespread over the past decade. However, the development of non-caseating granulomas with the use of monoclonal antibodies, such as ustekinumab, is not widely reported in the literature.

**Case presentation:**

We report a case of a 50-year-old Caucasian male who presented complaining of weight loss and shortness of breath. He was receiving ustekinumab for refractory psoriasis but had no other significant medical comorbidities. On physical examination, reduced breath sounds on the right side were noted. Blood cultures were drawn on presentation and came back negative in 48 hours. A chest computed tomography scan revealed a large right lung mass in addition to right-sided pleural effusion. Therapeutic thoracocentesis was done; fluid cytology and analysis were negative for malignancy, acid-fast bacilli, or fungal infections. A positron emission tomography scan showed multifocal radiotracer uptake including within right lung mass, multiple bones, lymph nodes, liver and spleen. Biopsies showed hyalinized non-necrotizing granulomas. Immunohistochemical stains for AE1/AE3, cytokeratin 7 and 20, and thyroid transcription factor 1, were all negative. He was started on steroid therapy, and ustekinumab was discontinued and the follow-up computed tomography after a few months showed substantial improvement. However, over the course of next 4 months patient developed hepatic dysfunction and recurrent ascites and ultimately underwent transjugular intrahepatic portosystemic shunt placement. Furthermore, he was started on azathioprine and steroids were tapered. He improved clinically and was discharged from our hospital within a week.

**Conclusions:**

This case highlights the need for careful consideration of patient medication history while evaluating the possible differential diagnoses that may contribute to a patient’s presentation.

## Introduction

Monoclonal antibody therapies have been frequently used in recent years in the treatment of chronic inflammatory disorders due to their distinctive immunosuppressive and selective properties. Ustekinumab is a human monoclonal antibody (mAb) designed to block interleukin (IL)-12 and IL-23 from binding to their p40 Beta subunit receptors on the surface of T and natural killer (NK) cells [1], thereby neutralizing intracellular phosphorylation, preventing cytokine production, inhibiting molecular expression, and, ultimately, dysregulation of the Th1 and Th17 pathways.

Ustekinumab is Food & Drug Administration (FDA) approved to treat moderately severe Crohn’s disease, moderate or severe plaque psoriasis, and active psoriatic arthritis in adults, given its revolutionary benefit in treating chronic inflammatory disorders. Common side effects of ustekinumab include infections, allergic reactions, and gastrointestinal upset. Rarely, it can lead to delayed cutaneous reactions [2]. Previously reported cases had suggested a possible correlation between the administration of ustekinumab and the development of non-caseating granulomas in different organs.

Our case demonstrates the potential inducement of multi-organ lesions as evidenced by imaging when being treated with ustekinumab for psoriasis.

## Case presentation

**Fig. 1:**
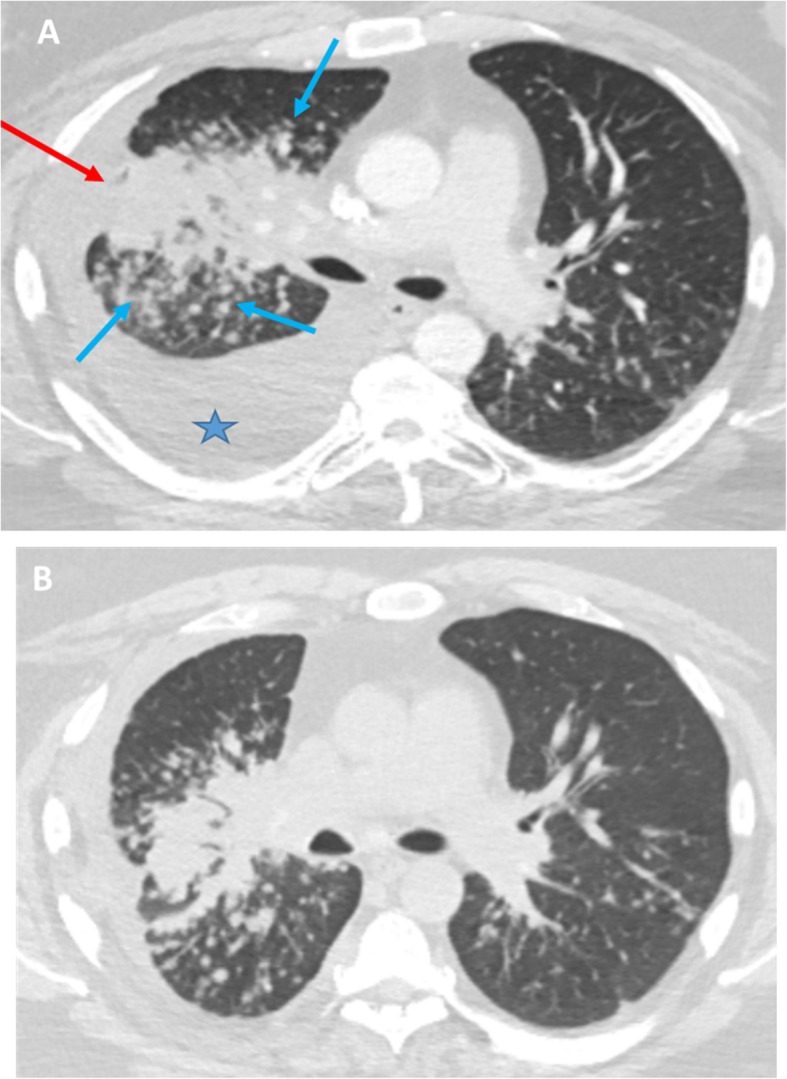
**a** Axial chest computed tomography image at presentation demonstrating a large area of right mid lung consolidation (red arrow) with adjacent extensive nodularity (blue arrows) along with a large right plural effusion (star). **b** Follow up Chest computed tomography after 8 months of prednisone administration results in significant improvement of right lung infiltrates as well as resolution of right sided pleural effusion

**Fig. 2:**
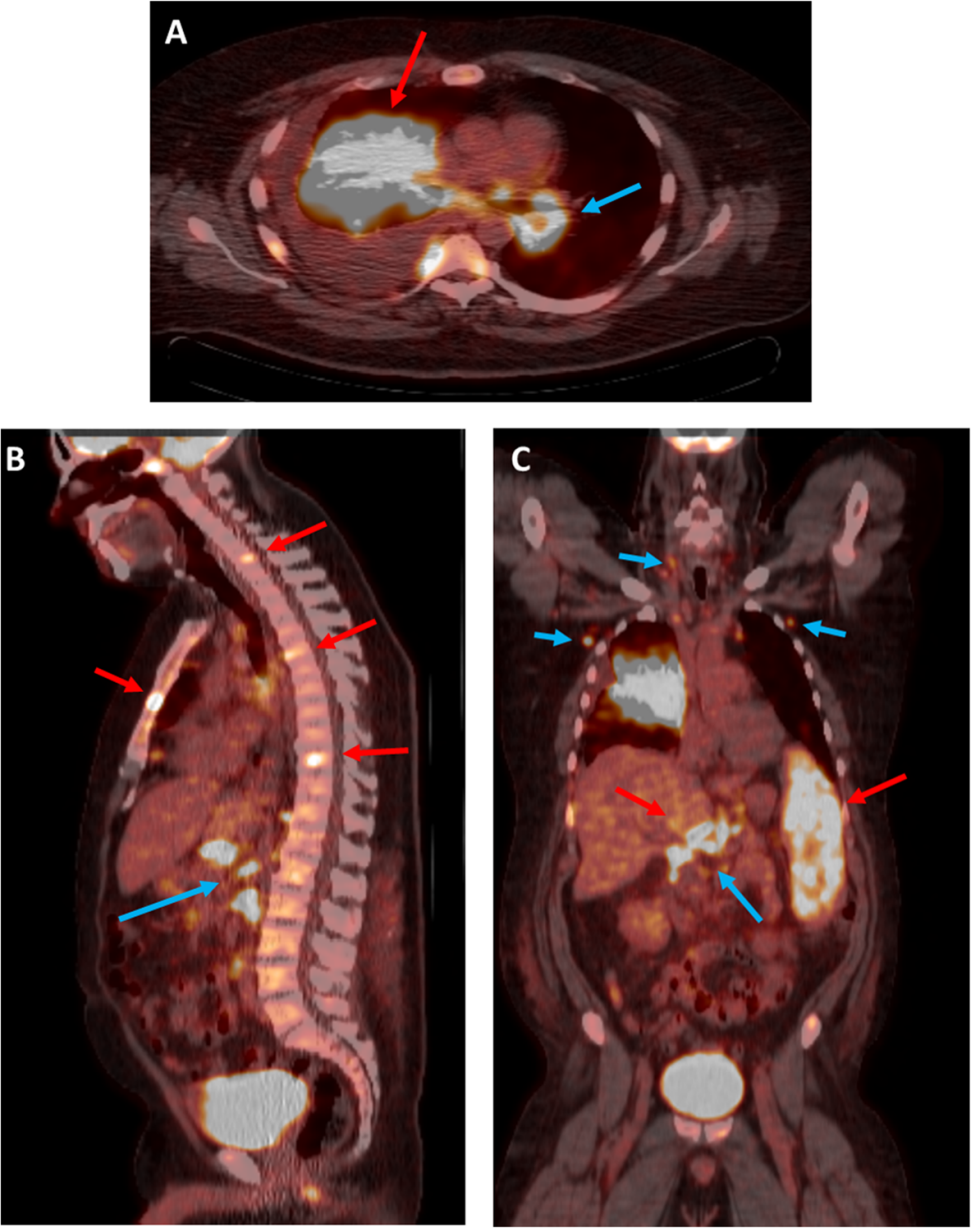
Axial, sagittal and coronal fused PET-CT images demonstrating widespread radiotracer uptake, including within right lung mass (red arrow in **a**), liver, spleen, multiple bones (red arrows in **b** and **c**) and multiple lymph node stations in lower neck, chest and upper abdomen (blue arrows in **a**, **b** and **c**)

A 50-year-old Causcasian man with a known history of refractory psoriasis on treatment with ustekinumab presented with a complaint of significant weight loss and shortness of breath. He had no other complaints. He denied a previous similar episode and any maculopapular rash or urticarial reaction after taking ustekinumab. He was fatigued but recalled no chest pain, palpitations, night sweats, cough, or recent infections. He had no other medical comorbidities or surgical history. A physical examination showed significant wasting but not acute distress. He had decreased breath sounds on the right side of his chest, but, otherwise, the physical examination was unremarkable. Blood cultures were drawn on presentation and came back negative in 48 hours. A chest computed tomography (CT) scan was performed and revealed a large right lung mass with adjacent nodularity in addition to right-sided pleural effusion (Fig. 1a), and possibility of primary lung malignancy was raised. Therapeutic thoracocentesis was done; fluid cytology and analysis were negative for malignancy, acid-fast bacilli, or fungal infections. A positron emission tomography (PET) scan was performed to complete the work up, which revealed multifocal areas of hypermetsabolic activity, including intense activity within right lung mass, nodular uptake in axial and visualized proximal appendicular skeleton, multiple lymph node groups in the lower neck, chest and upper abdomen, and diffuse uptake within liver and spleen (Figs. 2a, b, c). Disseminated malignancy was highly suspected at this point; thus, a transbronchial biopsy was done which showed respiratory mucosa with poorly formed non-necrotizing granulomas, and a right parietal pleura biopsy demonstrated non-necrotizing and hyalinized granulomatous inflammation. A left iliac bone biopsy was also obtained and showed benign bone tissue and bone marrow growth. Interventional radiology attempted to obtain a sample of the liver lesion, but the attempt was unsuccessful, because of poor viaualization of lesions on ultrasound. Immunohistochemical stains for AE1/AE3, cytokeratin 7 and 20, and TTF-1 were all negative. So, given the patients history of medical therapy and histopathological findings, a diagnosis of ustekinumab associated steroid reaction was felt to be most likely.

As a result, our patient was started on oral prednisone therapy, and ustekinumab was discontinued. Follow up CT of his chest demonstarted significant decrease in size of right lung mass and adjacent nodularity, in response to the prednisone (Fig. 1b). During the stroid taper, the patient developed hepatic dysfunction and portal hypertension as evidenced by recurrent pleural effusions and ascites. Diagnostic and therapeutic thoracocentesis was done and 2 liters of fluid was drained. A transjugular core biopsy of his liver was successful at that time and showed multiple necrotizing and non-necrotizing giant cell granulomas. The decision was made to start him on azathioprine, avoid anti-tumor necrosis factor (TNF) or mAb agents as well as methotrexate due to its hepatic side effects, and to slowly taper his prednisone dosage with subsequent follow-up in our out-patient clinic. Despite this, in next two months the hepatic dysfunction continued to deteriorate and patient had to undergo TIPS( transjugular intrahepatic portosystemic shunt) placement.

## Discussion

We report a rare case of disseminated sarcoid-like reaction in a patient undergoing treatment with ustekinumab for refractory psoriasis. Ustekinumab has a long history of successfully controlling chronic inflammatory arthropathies such as psoriasis and psoriatic arthritis [3], but significant unexpected sarcoid-like reactions with non-caseating giant cell granulomas have been reported in rare case reports. The overstimulation of cytokines, such as IL-12, IL-18, IL-27, and interferon (IFN)-gamma, has been postulated in lung sarcoidosis pathogenesis [4].

On the contrary, our patient was prescribed ustekinumab, a human mAb which is an anti-IL-12 blocker and anti-IL-23 blocker, for the treatment of refractory psoriasis and developed multiple non-caseating granulomas in his lung, liver, spleen and bones as evidenced by serial PET scan. This phenomenon is not entirely understood given the induction of sarcoid-like lesions while blocking the Th1 pathway. Furthermore, our patient was not-adherent to ustekinumab for his psoriasis adding to the complexity of the diagnosis.

Steroid use improved the lesions in his right lung, but given its widespread side effects with long term use and concurrent hepatic derangement, an immunosuppressive agent, azathioprine, was initiated due to its safety profile and ability to reduce intracellular purine synthesis leading to a decreased number of circulating T lymphocytes in an attempt to control the sarcoid-like lesions [5]. Review of previous case reports showed  the ability of ustekinumab to cause severe and harmful reactions. In addition to that, other similar immunosuppressive agents, like, adalimumab, have been implied in sarcoid-like reactions [6].

It is of great importance to carefully select the appropriate biological therapies when treating chronic inflammatory diseases given their recent emergence, clinical benefit, and unexpected side effects, which are evident in this article.

## Conclusions

In summary, this study presents a rare but significant case of sarcoid-like reaction due to immunomodulator medication therapy. The disseminated nature of the reaction resulted in our patient undergoing extensive invasive procedures to exclude a malignancy diagnosis. Physicians should pay close attention to medication history when interviewing patients and consider possible medication side effects while ordering invasive testing.

## Data Availability

Not available.
